# Characteristics of Low-Frequency Metasurface Microwave Absorption Filter with Composite Molding and Size Dependency

**DOI:** 10.3390/ma18225094

**Published:** 2025-11-10

**Authors:** Sangwon Baek, Wonwoo Choi, Sun-Woong Kim, Minah Yoon, Taein Choi, Hak Joo Lee, Kichul Kim

**Affiliations:** Center for Advanced Meta-Materials, 156-Gajengbuk-ro, Yuseong-gu, Daejeon 34103, Republic of Korea; bsangwon0425@camm.re.kr (S.B.); wwchoi@camm.re.kr (W.C.); woongskim1@naver.com (S.-W.K.); yoonma91@gmail.com (M.Y.); cti29@camm.re.kr (T.C.); hjlee@camm.re.kr (H.J.L.)

**Keywords:** microwave, low frequency, metasurface, absorption filter

## Abstract

This study presents a lightweight metasurface microwave absorption filter (MMAF) designed for low-frequency stealth applications in the L-band. The metasurface is optimized using a genetic algorithm to achieve broadband absorption at subwavelength thickness. The fabricated MMAF consists of a glass fiber-reinforced plastic layer, a metasurface layer, and a dielectric layer with a size of 30 × 30 cm^2^. It maintains a reflection coefficient below −10 dB in the frequency range of 1.01–1.78 GHz. Experiments using small MMAFs measuring 15 × 15 cm^2^ confirmed stable performance after composite molding. In addition, the results from small MMAFs configured in a 2 × 2 array were similar to those of the 30 × 30 cm^2^ structure. These results highlight the potential of the MMAF for scalable deployment on curved or segmented surfaces, expanding its applicability to various stealth platforms.

## 1. Introduction

Electromagnetic wave absorbers have become key for modern stealth systems and for addressing electromagnetic compatibility problems. As radar detection systems evolve toward multiband and long-range tracking capabilities, maintaining low observability across extended frequency bands has become a pressing challenge in stealth design [1,2,3,4,5,6,7]. In particular, L-band (1–2 GHz) absorbers are attracting increasing attention because of their relevance in long-range early warning systems and surveillance radars. With decreasing operating frequency, the absorber becomes thicker and heavier. Therefore, thin and lightweight metasurface absorbers are emerging as promising technologies [8,9,10,11,12,13,14,15,16].

Recently, several types of metasurface absorbers targeting the L-band have been introduced to overcome the thickness and weight limitations of absorbers. Frequency-selective surface (FSS)-based absorbers selectively absorb incident electromagnetic waves using periodic metallic patterns that resonate at specific frequencies [17,18,19,20,21,22,23]. Greater than 90% absorption across 1 to 2.51 GHz can be achieved using an ultra-miniaturized FSS absorber [24]. However, owing to their reliance on patterned metallic layers, FSS absorbers often require rigid substrates. This structural rigidity restricts mechanical flexibility and limits their integration onto lightweight or nonplanar surfaces.

Furthermore, magnetic materials with high magnetic loss are employed in metasurface absorbers to achieve strong absorption even at relatively thin thicknesses [25,26,27,28,29,30,31]. A multilayered magnetic absorber is reported to provide more than 90% absorption between 1.13 and 4.36 GHz at a total thickness of 13 mm [32]. Although this thickness is relatively small, the high density of magnetic materials results in a significant increase in weight and limits their use for lightweight platforms.

On the other hand, L-band absorbers can be designed using three-dimensional metamaterials with structural elements arranged in the vertical and horizontal directions [33,34,35,36,37]. A three-dimensional fractal tree metamaterial absorber achieved 80% absorption from 0.7 to 2.0 GHz with a compact thickness of 24 mm [38]. However, the structural stability of these absorbers is not considered, which makes them unsuitable for practical applications.

Moreover, lumped resistors can be integrated into metasurface absorbers to tailor their absorption characteristics [39,40,41,42,43,44,45]. An absorption of more than 90% is observed between 0.78 and 2.04 GHz for a structure that combines magnetic materials and a 62 Ω lumped resistor [46]. This type of structure has obvious limitations in practical applications because of parasitic reactance and detuning effects for broadband operation.

Such problems can be solved by tailoring surface impedance by optimizing binary-coded metasurface patterns on flexible substrates. This approach yields acceptable absorption in the low-frequency regime and offers the potential for integration with curved or segmented composite structures. In this paper, a lightweight metasurface microwave absorption filter (MMAF) optimized for low-frequency applications in the L-band is proposed. The metasurface pattern is encoded as a binary matrix and optimized through a genetic algorithm [47] to ensure broadband absorption under subwavelength thickness. The MMAF is fabricated by screen-printing resistive pixel patterns on a flexible polyimide (PI) film. Subsequently, the printed layer is laminated with a glass fiber-reinforced plastic (GFRP) layer and a dielectric foam through a composite molding process. The electromagnetic performance is measured for both non-molded and molded samples. The effect of sample size on absorption performance is evaluated by comparing large-scale and small-scale configurations.

## 2. Design of Metasurface Microwave Absorption Filter (MMAF)

The MMAF operates via destructive interference between the incident and PEC-reflected waves [48,49]. In a PEC-backed stack with a thin and resistive metasurface, the spacer thickness *t* is set by

(1) $$t=\frac{\left(2n-1\right)\lambda_{d}}{4}$$

where *λ*_d_ is the wavelength in the spacer. Under this condition, the phase accumulated in the spacer together with the π inversion upon PEC reflection sums to $\left(2n-1\right)\pi$, which establishes the *n*-th interference resonance at

(2) $$f_{n}=\frac{\left(2n-1\right)c}{4n_{eff}t},\;\;\;\;n_{eff}\approx \sqrt{\epsilon_{r}^{\prime }}.$$

The thickness of the spacer is determined based on the first harmonic frequency *f*_1_, which lies in the L-band. The pixel-based metasurface pattern is then used to realize the required loss distribution for this thickness.

**Figure 1:**
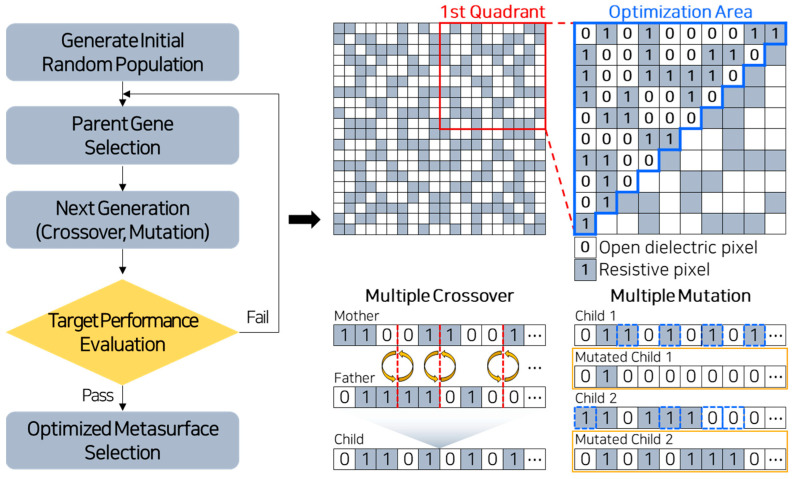
Algorithmic framework of the genetic algorithm.

Accordingly, to realize a lightweight and thin MMAF, the pixel-based metasurface is optimized through a genetic algorithm (GA) [47]. The algorithmic framework is shown in Figure 1. A metasurface unit cell is encoded as a binary matrix with 20 × 20 square pixels, and each pixel is 1.5 × 1.5 mm^2^ in size. To reduce computational cost, only 55 pixels within half of the 1st quadrant of the unit cell are used as optimization variables. The full pattern is then constructed by applying diagonal, horizontal, and vertical symmetry operations. The constructed pattern is represented by a binary-coded chromosome in the GA. Each pixel represents either a resistive or dielectric domain, corresponding to the ‘1’ or ‘0’ pixels in the figure.

The algorithm begins by generating an initial population of binary-coded chromosomes. The next generations are then created through multiple operations of crossover and mutation from the chromosomes. Next, each chromosome in the next generation is evaluated to identify the optimal chromosome. The objective function S for the evaluation is as follows:

(3) $$S=\frac{1}{N}\sum_{n=1}^{N}W_{n}\cdot \left|\Gamma \left(f_{n}\right)\right|$$

where Γ ($f_{n}$) denotes the reflection coefficient at the n-th frequency point, W_n_ is a weight factor, and N is the number of frequencies. The reflection coefficient of the MMAF is calculated using COMSOL Multiphysics 6.2, a commercial finite element method simulation software. A perfectly matched layer is used on the top boundary to avoid the occurrence of reflected electromagnetic waves at the edge of the structure, and periodic boundary conditions are used to assume a uniform array of unit cells with a given periodicity.

**Figure 2:**
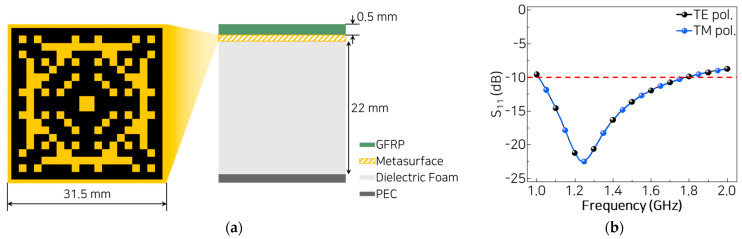
(**a**) Layered structure and unit cell layout of the proposed MMAF. (**b**) Simulated reflection losses of the MMAF under TE and TM polarizations.

The final configuration of the MMAF is illustrated in Figure 2a. The MMAF is composed of three layers: a GFRP layer, a metasurface layer, and a dielectric foam. A perfect electric conductor (PEC) is placed on the bottom of the MMAF as a reflector. The complex relative permittivity of the dielectric foam is 1.13–j0.0025 (Diab divinycell, F90), and that of the GFRP layer is 4.1–j0.082 (HG1581/RS1212). The thicknesses of the dielectric foam and the GFRP layer are 22 mm and 0.5 mm, respectively. The electrical conductivity of the resistive pixels is set to 3300 S/m. Accordingly, the total thickness of the MMAF is 0.076 *λ*_max_, where *λ*_max_ corresponds to 1 GHz, the lower edge of the L-band.

Given these considerations, the absorption characteristics of the optimized MMAF are assessed across the design frequency range. The simulated reflection performance of S_11_ remains below −10 dB from 1.01 to 1.78 GHz, confirming adequate operation within the L-band under normal incidence (Figure 2b). Reflection increases gradually beyond the design frequency band, as impedance matching deteriorates at higher frequencies. The response curves for both the transverse electric (TE) and transverse magnetic (TM) polarizations are indistinguishable across the frequency band, indicating polarization-insensitive behavior. This characteristic stems from the symmetric geometry of the pixelated unit cell, which provides matched surface impedance regardless of the polarization orientation. These results align with those of prior studies on metasurface absorbers, in which geometric symmetry has been shown to suppress polarization sensitivity and promote uniform field coupling [48,49].

## 3. Fabrication and Experimental Validation of MMAF

**Figure 3:**
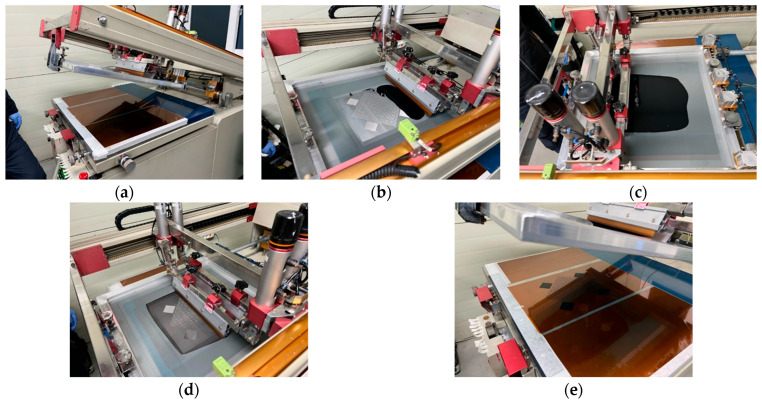
Screen-printing processes for metasurface fabrication. (**a**) PI film loading onto the printing stage. (**b**) Ink deposition on the mesh screen. (**c**) Uniform spreading of the ink. (**d**) Pattern transfer. (**e**) Final printed metasurface pattern on the PI film.

The metasurface layer of the MMAF is fabricated using a screen-printing process. The fabrication steps are illustrated in Figure 3. First, a flexible PI film with a thickness of 50 μm is loaded under the screen (Figure 3a). Subsequently, a resistive ink is deposited onto the screen and spread uniformly across the surface using a scraper (Figure 3b,c). A squeegee is subsequently used to press the ink through the mesh openings on the screen (Figure 3d). Finally, the designed pattern is transferred onto the PI film. The electrical conductivity is evaluated using a four-point probe method on 45 × 45 mm^2^ square patches located on each side of the printed metasurface.

**Figure 4:**
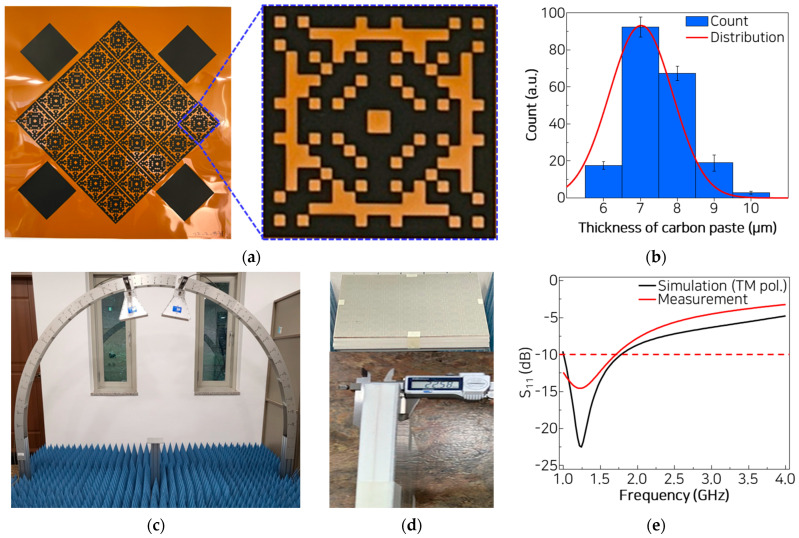
(**a**) Metasurface fabricated using screen printing. (**b**) Measured thickness distribution of the ink. (**c**) Measurement setup based on the NRL Arch system. (**d**) Thickness measurement of the non-molded MMAF. (**e**) Comparison between simulated and measured reflection losses for TM polarization.

The metasurface layer fabricated using screen printing is shown in Figure 4a, which reveals that the designed pixel pattern is precisely transferred onto the PI film and subsequently cured to ensure pattern stability. The thickness of the printed resistive pixels is measured using a digital dial gauge at 200 points for each of the five metasurfaces, and the average and standard deviation are calculated. The printed pixels exhibit a relatively uniform thickness distribution, with an average thickness of 7.49 µm. The standard deviation is 0.84 µm, which corresponds to approximately 11.2% of the mean thickness. This deviation can be further improved through refinement of the screen-printing process.

To verify the electromagnetic wave absorption performance of the MMAF prior to composite molding, a non-molded MMAF is fabricated by sequentially stacking the individual layers of the proposed structure. The dimensions of the fabricated MMAF are 30 × 30 cm^2^. Each layer is assembled manually without application of heat or mechanical pressure, and the edges are secured using adhesive tape to retain structural alignment during measurement.

The non-molded MMAF is then evaluated using the Naval Research Laboratory (NRL) arch measurement method (Figure 4c). In this setup, two broadband horn antennas are used to transmit and receive electromagnetic waves. The antennas are mounted along the arch frame. Due to physical interference between the antennas, the minimum incident angle of the measurement is limited to ±10°. These antennas are connected to a vector network analyzer (Keysight Technologies E5080B, Keysight Technologies, Santa Rosa, CA, USA) and operated in the frequency range of 1 to 18 GHz. A sample stage is positioned at the center of the arch between the two antennas, where the microwave transmitted from port 1 is reflected by the sample and subsequently received by port 2. Pyramidal microwave absorbers are placed on the floor of the system to minimize the reflected waves from the floor. Reflection from the back wall is not significant; this can be found from the signal response in the time domain. In addition, a time-gating technique is applied to suppress spurious reflections from unintended paths as well as coupling between the two antennas. The reflection performance of the sample is obtained by normalizing the measured reflection with respect to a metallic plate as a reference. The metallic plate of the same size as the sample is first measured to acquire the reference reflection, followed by the measurement of the sample under identical conditions. By comparing the two reflection signals, the intrinsic absorption characteristics of the sample are extracted while eliminating system and environmental effects.

The thickness of the non-molded MMAF is measured using a digital Vernier caliper at 22.58 mm, which matches the design thickness of 22.5 mm used in the simulation. The measured reflection losses are compared with the simulated losses in Figure 4e. Considering the polarization-insensitive nature of the MMAF, only the TM polarization is evaluated in this comparison. The lowest reflection is observed at 1.25 GHz from the simulation and at 1.23 GHz from the measurement. This small difference indicates that the metasurface pattern is accurately reproduced in the printing process. This also implies that the electrical conductivity of the designed MMAF is acceptable.

The measured S_11_ of the MMAF exhibited a reflection bandwidth below −10 dB from 1.00 to 1.71 GHz, which is slightly lower than the simulated value from 1.01 to 1.78 GHz. Considering that the measurement system does not support frequencies below 1.0 GHz, the actual absorption band is likely to be wider. This suggests that the simulation and measurement results are in good agreement.

**Figure 5:**
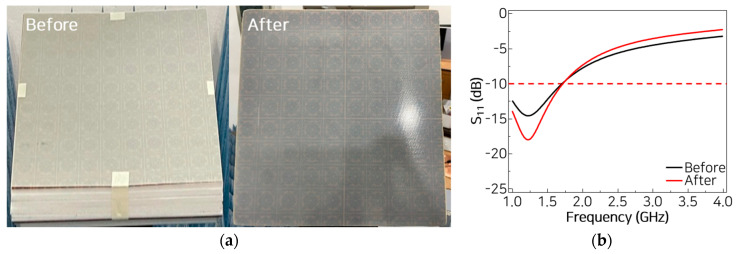
(**a**) Photographs and (**b**) measured reflection losses of the MMAF before and after composite molding.

To evaluate the structural and electromagnetic effects of composite molding, a hot-pressing process is performed on MMAF structures with dimensions of 30 × 30 cm^2^ at 130 °C and 3 bars. Figure 5a shows photographs of the MMAF before and after composite molding, where the overall structure is visually preserved.

The reflection losses of the molded MMAF are subsequently evaluated and compared with those of the non-molded MMAF. Both the lowest reflection and the reflection bandwidth below −10 dB remain at the same levels before and after composite molding. These findings confirm that the electromagnetic wave absorption performance of the MMAF is not affected significantly by thermal or mechanical compression.

## 4. Evaluation of Size-Dependent Absorption Performance

**Figure 6:**
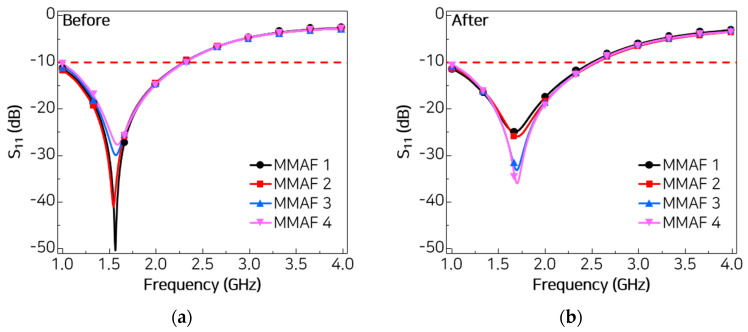
Measured reflection losses of four small-scale MMAFs (**a**) before and (**b**) after composite molding.

To investigate the consistency of the absorption performance of the MMAF in reduced-size applications, four small-scale MMAFs measuring 15 × 15 cm^2^ are fabricated. Each MMAF is subjected to composite molding under the same conditions described above. The reflection losses of the MMAFs are measured before and after composite molding, and a comparison is presented in Figure 6. The figure shows that the lowest reflection frequency of all four MMAFs shifted upward after composite molding, and the reflection bandwidth below −10 dB also broadened. The results are summarized in Table 1. The observed shifts range from 6.9% to 9.7% for the lowest reflection frequencies and from 8.7% to 11.3% for the bandwidths. This discrepancy can be attributed to the difference in time gating conditions during measurement caused by the small sample size.

**Figure 7:**
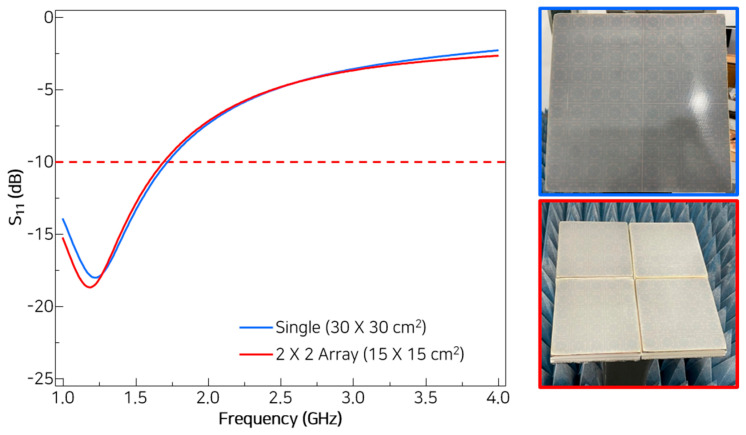
Comparison of measured reflection losses for a single large-scale MMAF and a 2 × 2 array of small-scale MMAFs.

To examine the feasibility of using arrayed small MMAFs as an alternative to large-scale MMAFs, a 2 × 2 array configuration is assembled using four small-scale MMAFs. The measured reflection losses of the 2 × 2 array are very close to those of the single large-scale MMAF. In both configurations, the MMAFs exhibit the lowest reflection frequency of approximately 1.2 GHz and almost identical reflection bandwidths below −10 dB, as shown in Figure 7.

## 5. Discussion

In Table 2, the performance of MMAF is compared to that of the absorbers reported in recent years. The proposed MMAF is lightweight, thin, and practical. The dielectric foam with the screen-printed metasurface of this study avoids the mass penalties of magnetically loaded absorbers. It also offers greater structural robustness than absorbers based on air spacer or honeycomb core. In addition, fabrication in this study relies on large-area screen printing followed by composite molding. This route removes photolithography, etching, metallization, discrete-resistor placement, or the 3D printing process. As a result, the process cost per area is lowered and production of large panels is possible. Additionally, usability is supported by the evaluation of size-dependent absorption performance. The absorption performance is essentially unchanged before and after composite molding. The design therefore supports conformal installation on moderately curved or segmented structures. Small tiles (e.g., 15 × 15 cm) assembled into larger panels reproduce the response of a single large panel. This enables modular coverage and field-level repair.

## 6. Conclusions

This study demonstrates a lightweight and thin MMAF for low-frequency stealth applications in the L-band. The MMAF is designed using a genetic algorithm to optimize the absorption performance of the metasurface. The metasurface achieved polarization-insensitive absorption with a reflection bandwidth of −10 dB from 1.01 to 1.78 GHz. An MMAF 30 × 30 cm^2^ in size is fabricated using screen printing and subsequently integrated into composite molding through a hot-pressing process. Experimental measurements confirm that the absorption is close to the calculated results even after composite molding. Additional evaluations with small-scale MMAFs show that the absorption performance is preserved even when the structure is separately molded and then assembled. These results demonstrate that the proposed MMAF can be applied to areas where full-size MMAF integration is often limited because of structural constraints. Furthermore, these findings verify the potential of the small-sized MMAF for use in a wide range of military platforms requiring low-frequency stealth capabilities.

## Figures and Tables

**Table 1:** Lowest reflection frequency and −10 dB bandwidth of MMAFs.

MMAF	Composite Molding	Lowest Reflection Frequency (GHz)		−10 dB Bandwidth (GHz)	
1	Before	1.56	+7.1%	1~2.3	+8.7%
	After	1.67		1~2.5	
2	Before	1.55	+9.7%	1~2.3	+11.3%
	After	1.7		1~2.56	
3	Before	1.57	+8.3%	1~2.31	+10.4%
	After	1.7		1~2.55	
4	Before	1.59	+6.9%	1~2.32	+9.5%
	After	1.7		1~2.54	
Average		+8%		+9.98%	

**Table 2:** Comparison of low-frequency (1–2 GHz) absorbers.

Reference	−10 dB Bandwidth (GHz)	Spacer	Type	Thickness (*λ*_max_)
[24]	1–2.51	Magnetic	FSS + Resistor	0.012
[32]	1.13–4.36	Magnetic	Multilayer stacking	0.049
[38]	0.7–2	-	3D structure + Resistor	0.056
[46]	0.78–2.04	Magnetic	FSS + Resistor	0.024
[50]	1–2.74	Air	FSS + Resistor	0.078
[51]	1–4.5	Honeycomb	FSS	0.088
This work	1.01–1.78	Dielectric Foam	Metasurface	0.076

## Data Availability

The original contributions presented in this study are included in the article. Further inquiries can be directed to the corresponding author.

## Abbreviations

The following abbreviations are used in this manuscript:

MMAF	Metasurface Microwave Absorption Filter
GA	Genetic Algorithm
PI	Polyimide
GFRP	Glass Fiber-Reinforced Plastic
PEC	Perfect Electric Conductor
TE	Transverse Electric
TM	Transverse Magnetic

## References

[1] Zohuri B. (2020). Radar Energy Warfare and the Challenges of Stealth Technology.

[2] Ray S., Panwar R. (2024). Advances in Polymer-Based Microwave Absorbers-From Design Principles to Technological Breakthroughs: A Review. IEEE J. Flex. Electron..

[3] Zhang Y., Gong X., Zeng Y., Dong X., Guo H., Jiang Z., Hu T., Wang J., Zou Y., Chu Z. (2025). An Urchin-Inspired Broadband and Ultralight Microwave Absorber. Adv. Funct. Mater..

[4] Duong T.X., Tung D.K., Pham T.S., Nguyen H.A., Tung B.S., Hai N.P., Hanh V.T.H., Dat T.Q., Khuyen B.X., Lam V.D. (2025). Broadband metamaterial absorber in the C-Ku bands by exploiting FeCo-C. J. Appl. Phys..

[5] Yan M., Pan Y., He P., Gong L., Fu Y., Zhang H., Cheng X. (2025). Flexible Hierarchical Hollow SiC/SiO_x_ Micro/nanofiber Sponges for Broadband Electromagnetic Wave Absorption. Adv. Fiber Mater..

[6] Luo F., Liu D., Cao T., Cheng H., Kuang J., Deng Y., Xie W. (2021). Study on broadband microwave absorbing performance of gradient porous structure. Adv. Compos. Hybrid Mater..

[7] Hao J., Zhang B., Jing H., Wei Y., Wang J., Qu Z., Duan J. (2022). A transparent ultra-broadband microwave absorber based on flexible multilayer structure. Opt. Mater..

[8] Singh G., Bhardwaj A., Srivastave K.V., Ramkumar J., Ramakrishna S.A. (2021). Perforated lightweight microwave metamaterial broadband absorber with discontinuous ground plane. Appl. Phys. A.

[9] Kang J., Qu Z., Duan J., Jing H., Hao J., Song C., Wang J., Zhang B. (2023). Multispectral flexible ultrawideband metamaterial absorbers for radar stealth and visible light transparency. Opt. Mater..

[10] Tirkey M.M., Gupta N. (2021). A Novel Ultrathin Checkerboard Inspired Ultrawideband Metasurface Absorber. IEEE Trans. Electromagn. Compat..

[11] Qu Z., Hao J., Jing H., Wei Y., Duan J., Wang J., Zhang B. (2022). An ultra-thin ultra-broadband microwave absorber for radar stealth. Adv. Compos. Hybrid Mater..

[12] Dong F.Y., Niu C., Zhang M., Wang A., Duan K., Zhao J., Zhu W., Hou Z. (2025). A Lightweight Ultra-Wideband Metasurface Microwave Absorber. Adv. Mater. Technol..

[13] Feng M., Zhang K., Xiao J., Liu B., Cheng H., Li Y., Zhao Z., Liang B. (2023). Material-structure collaborative design for broadband microwave absorption metamaterial with low density and thin thickness. Compos. Part B Eng..

[14] Liu L., Chen Z., Li Z., Chang Y., Li P., Liu X., Deng X., Feng Y. (2025). Design of a Low-Infrared-Emission and Wideband-Microwave-Absorption Lightweight Metasurface. Nanomaterials.

[15] Guo M., Wang X., Liu Y., Liu H., Zhou K., Wang J., Qu S., Liu S. (2025). Enhancing low-frequency performance of thin-layer magnetic microwave absorbing materials via phase gradient metasurface. Mater. Des..

[16] Chen N., He C., Zhu W. (2023). Lightweight Machine-Learning Model for Efficient Design of Graphene-Based Microwave Metasurfaces for Versatile Absorption Performance. Nanomaterials.

[17] Tennant A., Chamber B. (2004). A single-layer tuneable microwave absorber using an active FSS. IEEE Antennas Wirel. Propag. Lett..

[18] Panwar R., Puthucheri S., Singh D., Agarwala V., Lee J.R. (2016). Microwave absorption properties of FSS-impacted composites as a broadband microwave absorber. Adv. Compos. Mater..

[19] Agrawal A., Kumar A., Panwar R. (2025). Resistive ink derived FSS-based microwave absorber using equivalent circuit modelling-interfaced deep learning technique. Appl. Phys. A.

[20] Jorwal S., Dubey A., Gupta R., Agarwal S. (2023). A review: Advancement in metamaterial based RF and microwave absorber. Sens. Actuators A Phys..

[21] Zheng L., Yang X., Gong W., Qiao M., Li X. (2022). Ultralow Thickness-Bandwidth Ratio Magnetic Absorber with Printed FSS for S&C Bands. IEEE Antennas Wirel. Propag. Lett..

[22] He F., Si K., Zha D., Li R., Zhang Y., Dong J. (2021). Broadband Microwave Absorption Properties of a Frequency-Selective Surface Embedded in a Patterned Honeycomb Absorber. IEEE Trans. Electromagn. Compat..

[23] Liu Y., Yang Y., Xu J., Lu L., Su X. (2022). Electromagnetic and microwave absorption properties of Ti_3_SiC_2_/AgNWs/acrylic acid resin composite coatings with FSS incorporation. J. Alloys Compd..

[24] Liu Z., Liu S., Xu Z., Mei Z., Niu T. (2024). Realization of an ultra-thin absorber based on magnetic metamaterial working at L-band. Appl. Phys. Lett..

[25] Zhang H., Zhao Y., Zuo X., Huang H., Sun C., Fan Z., Pan L. (2023). Construction of chiral-magnetic-dielectric trinity composites for efficient microwave absorption with low filling ratio and thin thickness. Chem. Eng. J..

[26] Elmahaishi M.F., Azis R.S., Ismail I., Muhammad F.D. (2022). A review on electromagnetic microwave absorption properties: Their materials and performance. J. Mater. Res. Technol..

[27] Jiao Z., Huyan W., Yao J., Yao Z., Zhou J., Liu P. (2022). Heterogeneous ZnO@CF structures and their excellent microwave absorbing properties with thin thickness and low filling. J. Mater. Sci. Technol..

[28] Gao S., Chen L., Zhang Y., Shan J. (2021). Fe nanoparticles decorated in residual carbon from coal gasification fine slag as an ultra-thin wideband microwave absorber. Compos. Sci. Technol..

[29] Ren Z., Liu X., Su J., Liu Y.Q., Zou H., Tian J., Sun X., Du X., Yin H. (2023). Low-profile broadband microwave absorber based on magnetic coating and artificial electromagnetic structures. Chem. Eng. J..

[30] Sani Y., Azis R.S., Ismail I., Yaakob Y., Mohammed J. (2023). Enhanced electromagnetic microwave absorbing performance of carbon nanostructures for RAMs: A review. Appl. Surf. Sci. Adv..

[31] Meng X., Lei W., Yang W., Liu Y., Yu Y. (2021). Fe_3_O_4_ nanoparticles coated with ultra-thin carbon layer for polarization-controlled microwave absorption performance. J. Colloid Interface Sci..

[32] Shou H., Feng J., Qi B., Qiao L., Niu T., Mei Z. (2023). A wideband absorber working in the L- and S-bands based on magnetic materials. Appl. Phys. Lett..

[33] Zhang S., An Q., Li D., Chen K., Zhao J., Jiang T., Chen P., Liao W., Liu T., Feng Y. (2025). Multifunctional meta-absorber based on CB-PLA composite and magnetic materials for electromagnetic absorption and load-bearing capacity. Compos. Sci. Technol..

[34] Tang Y., He L., Liu A., Chen Z., Wang W., Xu H., Deng L. (2024). Lightweight and dual-peak H-pattern metamaterial absorber based on discontinuous dielectric media in the L-band range. Int. J. Mod. Phys. B.

[35] Dai S., Liao S.Y., Chen Q., Ma H.C., Zhang H.F. (2025). Theoretical study of tunable tripling octave frequency to ultra-wideband low-profile metastructure absorber based on brick splicing method. Mater. Des..

[36] Lin F., Yan Z., Wang P., Wang Y., Zhou H., Lu H. (2024). A low-profile metamaterial absorber with ultrawideband reflectionless and wide-angular stability. Def. Technol..

[37] Yang Z., Liang Q., Duan Y., Liu P., Wang X., Li D. (2023). Electromagnetic characteristics and 3D-printing realization of a lightweight hierarchical wave-absorbing metastructure for low-frequency broadband absorption. J. Alloys Compd..

[38] Li Q., Dong J., Li T., Cao X. Broadband Fractal Tree Metamaterial Absorber for P and L Bands Applications. Proceedings of the 2020 IEEE 3rd International Conference on Electronic Information and Communication Technology (ICEICT).

[39] Kim Y.J., Hwang J.S., Yoo Y.J., Khuyen B.X., Rhee J.Y., Chen X., Lee Y.P. (2017). Ultrathin microwave metamaterial absorber utilizing embedded resistors. J. Phys. D Appl. Phys..

[40] Wang Q., Cheng Y. (2020). Compact and low-frequency broadband microwave metamaterial absorber based on meander wire structure loaded resistors. AEU-Int. J. Electron. Commun..

[41] Du Z., Liang J., Cai T., Wang G., Deng T., Wu B. (2022). Designing an ultra-thin and wideband low-frequency absorber based on lumped resistance. Opt. Express.

[42] Wei Y., Chen Y., Li Y., Li F., Wu Q., Wang J., Li B., Zhang B. (2023). Ultra-broadband 3D Metamaterial Microwave Absorber Based on Split-Ring Structure Loaded with Resistors and Magnetic Material. J. Electron. Mater..

[43] Kalraiya S., Chaudhary R.K., Abdalla M.A. (2022). Resistor loaded wideband conformal metamaterial absorber for curved surfaces application. AEU-Int. J. Electron. Commun..

[44] Ardeshana M.A., Thakkar F.N., Domadia S.G. (2025). Composite structure design for broadband metamaterial absorption: Integrated nonlinearity and enhanced performance using lumped resistors. J. Appl. Phys..

[45] Qu Z., Jing H., Deng H., Wei Y., Kang J., Wu X., Mi B., Li R., Wang J., Duan J. (2023). Ultra-wideband electromagnetic interference suppression lightweight metamaterial absorber based on S/C/X frequency band. Compos. Hybrid Mater..

[46] Liu Z., Liu Z., Zhang J., Xu Z., Yuan Z., Mei Z., Niu T. (2023). Realization of an ultra-thin absorber with fragmented magnetic structure at L-, S-, and partial C-bands. J. Appl. Phys..

[47] Katoch S., Chauhan S.S., Kumar V. (2021). A review on genetic algorithm: Past, present, and future. Multimed. Tools Appl..

[48] Kim Y., Park P., Jo J., Lee J., Jeong L., Shin J., Lee J.H., Lee H.J. (2021). Ultrawideband electromagnetic metamaterial absorber utilizing coherent absorptions and surface plasmon polaritons based on double layer carbon metapatterns. Sci. Rep..

[49] Lee C., Kim K., Park P., Jang Y., Jo J., Choi T., Lee H. (2023). Ultra-Wideband Electromagnetic Composite Absorber Based on Pixelated Metasurface with Optimization Algorithm. Materials.

[50] Zhang G.W., Gao J., Cao X., Li S.J., Yang H.H. (2019). Wideband miniaturized metamaterial absorber covering L-frequency range. Radioengineering.

[51] Zha D., Dong J., Cao Z., Zhang Y., He F., Li R., He Y., Miao L., Bie S., Jiang J. (2020). A multimode, broadband and all-inkjet-printed absorber using characteristic mode analysis. Opt. Express.

